# RECENT ADVERSITY AND LIFE SATISFACTION AMONG CHINESE OLDER ADULTS: ROLE OF GENDER AND NEIGHBORHOOD COHESION

**DOI:** 10.1093/geroni/igac059.2310

**Published:** 2022-12-20

**Authors:** Yi Wang, Ying Ma, Man Guo, Li Wang

**Affiliations:** University of Iowa, Iowa City, Iowa, United States; University of Houston, Houston, Texas, United States; University of Iowa, Iowa City, Iowa, United States; Anhui Medical University, Hefei, Anhui, China (People's Republic)

## Abstract

A growing body of literature suggests that recent adversity impedes psychological wellbeing. Little is known about whether living in a cohesive neighborhood could buffer the negative consequences of adverse experience and whether such associations vary by gender. This study aims to examine the association of recent adverse events and perceived life satisfaction among Chinese older adults, and to explore the potential moderating role of gender and neighborhood cohesion. Data were from a cross-sectional study conducted with community-dwelling older adults aged 60 years and above in Anhui Province, China in 2014 (N=1,960). Recent adversity was measured by six events in the past two years (health decline, economic difficulty, loss of intimate people, loss of an important item, major life event, conflict with family or close friends/neighbors). Multilevel ordered logit regression with interaction term of adversity and neighborhood cohesion was performed. Models were further conducted on stratified samples to compare gender differences. Individuals experiencing any recent adverse event are 84% less likely (p=.008) to report a higher level of life satisfaction. Living in neighborhoods with better cohesion increased the likelihood of reporting better life satisfaction by 6% (p=.031). Modeling on the stratified samples showed that the above-mentioned significant relationships hold for male sample only. Recent adversity may negatively affect life satisfaction among Chinese older adults. Living in neighborhood with higher levels of cohesion could help buffer such negative influences, particularly for older men. Findings highlight the critical role of neighborhoods in combating the negative psychological consequences of adversity among Chinese older adults.

